# Mannose-binding lectin deficiency with eosinophilic meningoencephalitis due to *Angiostrongylus cantonensis *in children: a case series

**DOI:** 10.1186/1752-1947-5-330

**Published:** 2011-07-28

**Authors:** Bárbara Padilla-Docal, Alberto J Dorta-Contreras, Raisa Bu-Coifiu-Fanego, René H Martínez-Alderete, Olga Susana de Paula-Almeida, Hansotto Reiber, Jens Christian Jensenius

**Affiliations:** 1 Faculty of Medical Sciences, "Dr Miguel Enriquez" Central Laboratory of Cerebrospinal Fluid (LABCEL), Havana Medical Sciences University, AP 10049, 11000 CP Havana City, Cuba; 2 Neurochemistry Laboratory, Neurology Hospital, Georg August University, Göttingen, Germany; 3 Department of Medical Microbiology and Immunology, University of Aarhus, Aarhus, Denmark

## Abstract

**Introduction:**

Eosinophilic meningitis, a potentially fatal disease caused by *Angiostrongylus cantonensis*, is considered an emerging infectious disease.

**Case presentation:**

Three Caucasian boys (aged five-years-old, 10-years-old and six-years-old) with a diagnosis of eosinophilic meningoencephalitis caused by *Angiostrongylus cantonensis *were studied. Serum immunoglobulin A (IgA), IgM, IgG, and complements C3c and C4 levels were quantified by using an immunodiffusion technique. Immunoglobulin E in serum was quantified by nephelometry and mannose-binding lectin by time-resolved fluorometry. Mannose-binding lectin deficiency was observed in the three patients. The first patient showed a reduction in the levels of IgA and IgM and an increase in the values of IgE and C4. The second patient showed a reduction in mannose-binding lectin level with increased IgG, C4 and IgE levels, and the third patient showed a decrease in mannose-binding lectin level and increased levels of IgM and complement C3c as well as a low level of C4.

**Conclusions:**

To the best of our knowledge, this is the first report of mannose-binding lectin deficiency associated with *Angiostrongylus cantonensis *meningoencephalitis in children, and it may contribute to the understanding of the participation of this component of the lectin pathway in the development of the disease.

## Introduction

Eosinophilic meningitis, a potentially fatal disease caused by *Angiostrongylus cantonensis*, a parasitic nematode, is considered an emerging infectious disease [1]. Adult *A. cantonensis *live in the pulmonary arteries of its definitive hosts, that is, rodents, especially rats, which pass infective first-stage larvae (L1) in their feces. The life cycle also involves mollusks harboring larval stages. In humans, larvae fail to mature, and hence humans and their excreta play no role in the transmission and direct dissemination of the parasite. Humans become infected by ingesting third-stage larvae (L3) in raw or undercooked intermediate host mollusks (for example, snails and slugs) or paratenic hosts (for example, freshwater prawns, crabs, frogs and fish) [1]. Lettuce and vegetable juice have also been identified as sources of infection when contaminated with intermediate or paratenic hosts [1].

The complement system provides an important effector mechanism of innate immune defense. Activation of the complement system proceeds through three different pathways converging in the activation of complement C3. The classical pathway is typically initiated after antigen recognition by antibodies, the alternative pathway relies on interference by foreign substances in a delicate activation-inhibition balance and the third pathway, the mannan-binding lectin (MBL) or lectin pathway, is initiated when one of the molecules MBL, L-ficolin or H-ficolin recognizes ligands arranged in patterns characteristic of microbial surfaces, pathogen-associated molecular patterns or pathogen-associated molecular patterns [2].

The objective of the present paper is to present the association between MBL deficiency and meningoencephalitis due to *Angiostrongylus cantonensis *in three children. The research project was approved by the hospital ethical committee, and the written informed consent of each parent or guardian was obtained.

### Case presentations

Case 1 is a five-year-old Caucasian Cuban boy; suffering from eosinophilic meningoencephalitis caused by *Angiostrongylus Cantonensis *was admitted to the hospital in May 2005. The clinical and neuroimmunological diagnoses were performed according to the protocol described in an earlier publication [3]. For all measurements, aliquots of serum were frozen and kept at -20°C for further analysis.

Serum levels of immunoglobulin A (IgA), IgM and IgG were quantified by an immunodiffusion technique using NOR Partigen immunoplates purchased from Siemens (Marburg, Germany). The levels of IgE in serum were quantified by N Latex IgE Mono immunoassay in a BN Prospec nephelometer (Dade Behring). C3c and C4 were quantified by using an immunodiffusion technique employing C3c NOR Partigen and C4 NOR Partigen immunoplates (Siemens). A detailed description of buffers and reagents has been given elsewhere [4]. The assay is a variant of the assay described by MacDonald *et al *[5].

In brief; monoclonal anti-MBL antibody was coated on the surface of microtiter wells. Plasma samples diluted in a buffer consisting of 20 mM Tris, 1 M NaCl, 10 mM CaCl_2_, 0.05% (vol/vol) Triton X-100, 0.1% human serum albumin (wt/vol), heat-aggregated normal human IgG (10 mg/ml), 1% (vol/vol) bovine serum albumin, pH 7.4, was added to the wells. Following incubation, the wells were washed, and europium-labeled anti-MBL antibody was added. After another incubation and wash, enhancement buffer was added and the bound europium was measured by time-resolved fluorometry. Dilutions of a standard plasma as well as a sample of plasma with known high (1046 ng of MBL/ml), middle (251 ng of MBL/ml) and low (38 ng of MBL/ml) concentrations were included as internal controls. The inter-assay coefficients of variation (%) for the three concentrations calculated on the basis of 20 assays were 8%, 8% and 11%, respectively [4].

The clinical diagnosis of the illness was fever, vomiting and irritation, together with a cerebrospinal fluid analysis with the presence of eosinophils and high cellular countdown in this biological liquid. In Cuba, these characteristics are enough to diagnose this disease because there is no other agent that produces eosinophils in this biological fluid [6,7].

The individual concentrations in blood of the major immunoglobulins, IgE, C3c, C4 and MBL may be observed in Table 1. The values of each of these serum proteins were contrasted with the normal values reported for boys in this age group [8]. Normal values for MBL have been considered in previous reports [2].

**Table 1:** Serum immunoglobulin, complement components and MBL levels^a^

Patient	**Age**, years	IgA (g/l)	IgG (g/l)	IgM (g/l)	IgE (IU/ml)	C3c (g/l)	C4 (g/l)	MBL
1	5	0.32	13.58	1.21	70.6	0.39	0.52	30 ng/ml
Normal values	3 to 5	0.55-1.52	5.69-15.97	0.22-1	60	0.55-1.2	0.2-0.5	1.5 g/ml
2	10	0.54	20.36	0.93	39.9	0.27	0.59	390 ng/ml
Normal values	9 to 11	0.12- 2.08	7.79-14.56	0.35-1.32	200	0.55-1.2	0.2-0.5	1.5 g/ml
3	6	1.13	8.15	1.7	n.d.	2.18	0.41	385 ng/ml
Normal values	6 to 8	0.54-2.21	5.59-14.92	0.27-1.18	90	0.55-1.2	0.2-0.5	1.5 g/ml

^a^MBL, mannose-binding lectin; IgA, immunoglobulin A; IgG, immunoglobulin G, IgM, immunoglobulin M; IgE, immunoglobulin E; C3c, complement C3c; C4, complement C4; n.d., no data.

He has antecedents of having had other illnesses before the present disease, such as acute diarrhoea with a remarkable serum MBL deficiency. At the moment of the first diagnosis of lumbar puncture, the patient had a level of glucose/serum of 0.54, which shows that there was no a bacterial process. In the differential cell count, the lymphocytes were predominant at 77%, and the presence of eosinophils in the blood and CSF were typical for cases of the illness.

At the moment of the first diagnostic lumbar puncture, this patient had a combined IgA and IgM deficiency because of serum levels. On the other hand, his IgG levels were in the normal range and his IgE and C4 levels were increased.

According to the normal values that have been reported for the Cuban population, he showed a reduction of the values of IgA and IgM and an increase in the values of IgE and C4. His MBL levels were also reduced. This patient had only a reduction of MBL levels, and his C4 and IgE levels were increased. Increased serum IgE level is compatible with the reported presentation in this type of parasitic infection [9].

This presentation corresponds perfectly with previous reports in which IgE values were increased in blood in accord with this illness, where *A. cantonensis *larvae go first to the lungs of the patient and later to the brain throughout the blood [9].

The blood and CSF profiles of this patient are given in Table 2. The most significant infectious disorders associated with MBL deficiency that the patient experienced were observed in Table 3.

**Table 2:** Blood and CSF profiles

Patient	Blood eosinophils,%	Blood glucose, mM/l	CSF cell count, 10^-6^	CSF eosinophils,%	CSF glucose, mM/l
1	7%	2.35	857	32%	4.33
2	12%	5.9	402	45%	3.2
3	10%	5.9	630	31%	3.1
Normal rank					

^a^CSF, cerebrospinal fluid.

**Table 3:** Clinical features of patients suffering from *Angiostrongylus cantonensis *meningoencephalitis with MBL immunodeficiency

Patient	Clinical features
1	Viral diarrhea (age nine months), viral diarrhea (age 11 months) Eosinophilic meningoencephalitis (age five years), pneumonia and bronchitis (age five and one-half years)
2	Allergy, pneumonia (age six months), bacterial diarrhea (age one year), sepsis and dehydration (age six years), eosinophilic meningoencephalitis (age 10 years)
3	Toxocariosis (age two years), eosinophilic meningoencephalitis (age six years)

^a^MBL, mannose-binding lectin.

After helmintic meningoencephalitis, the patient kept on with a respiratory infection history with possible remains not related to *A. cantonensis *previous infection.

Case 2 is a 10-year-old Caucasian Cuban boy; suffering from eosinophilic meningoencephalitis caused by *Angiostrongylus Cantonensis *was admitted to the hospital in June 2008. The clinical and neuroimmunological diagnoses were performed according to the protocol described in an earlier publication [3]. For all measurements, aliquots of serum were frozen and kept at -20°C for further analysis. Immunoglobulins, C3c, C4 and MBL in CSF and serum analysis were determined as case 1.

The clinical diagnosis of the illness was fever, vomiting and headache, together with a cerebrospinal fluid analysis with the presence of eosinophils and high cellular countdown in this biological liquid. His concentrations in blood of the major immunoglobulins, IgE, C3c, C4 and MBL may be observed in Table 1

This patient had the typical signs and symptoms of a meningoencephalitis. It was later diagnosed as eosinophilia because the high count of these cells in the CSF. This patient has a CSF/serum glucose quotient of 0.54, like a nonbacterial infection. Ninety percent of lymphocytes may be confused with viral meningoencephalitis if the presence of eosinophils in CSF is taken into account. As the previous case, this patient is characterized by having reduced MBL levels. It is compatible with a primary MBL immunodeficiency.

This patient had previously a recurrent infection that caused bacterial diarrhoea and some sepsis symptoms as well as dehidratation. During the illness produced by *A. cantonensis*, he had severe brain edema.

This patient showed a reduction of the values of C3c and an increase in the values of IgG and C4. IgA, IgM and IgE were normal. These figures are complemented by an increment of C4 levels. As in case 1, this boy had an MBL deficiency.

The blood and CSF profiles of this patient are given in Table 2. Table 3 shows the most significant infectious disorders associated with MBL deficiency that the patient experienced. The clinical features were obtained from their records.

Case 3 is a six-year-old Caucasian Cuban boy who was clinically diagnosed with eosinophilic meningoencephalitis on the basis of the symptoms typically observed in this illness, together with serum and CSF eosinophils. He was admitted to the hospital in May 2009. The clinical and neuroimmunological diagnoses were performed according to the protocol described in an earlier publication [3]. For all measurements, aliquots of serum were frozen and kept at -20°C for further analysis. Immunoglobulins, C3c, C4 and MBL in CSF and serum analysis were determined as case 1.

The clinical diagnosis of the illness was fever, vomiting and headache, together with a cerebrospinal fluid analysis with the presence of eosinophils and high cellular countdown in this biological liquid. Concentrations in blood of the major immunoglobulins, IgE, C3c, C4 and MBL may be observed in Table 1. He had a 0.52 CSF/serum glucose quotient with 87% lymphocytes.

This patient had a decrement of MBL but combined with normal IgA, IgG, and C4 values. MBL was found to be reduced, and his IgM and C3c were increased. During the acute phase, CSF and blood were taken for diagnostic purposes.

The increased C3c levels could ensure at least the functioning of the alternative pathway and part of the classical pathway. The increment of IgM values and the normal IgG values help to fix the complement system.

This patient had had parasitic infections of toxocariosis as well as after the eosinophilic meningoencephalitis diagnosis. He has not come back to the hospital for further treatment. This does not necessarily mean that he has completely recovered or has not had other infections; maybe he has gone to another medical facility for treatment.

The blood and CSF profiles of this patient are given in Table 2. His clinical features were obtained from their records. Table 3 shows the most significant infectious disorders associated with MBL deficiency that the patient experienced.

## Discussion

Functional MBL is a multimeric protein of up to six 96 kDa subunits, each consisting of three identical polypeptide chains produced by the liver. MBL recognizes mannose- and *N*-acetylglucosamine-rich oligosaccharides present on a wide range of bacteria, viruses, fungi and parasites. MBL interacts with mannose-associated serine proteases (MASP-1 and MASP-2), activates both the classical and alternate pathways of the complement system and may also bind to novel phagocyte receptors, resulting in opsonization, phagocytosis and cell lysis [10,11].

Our patients have very low levels of MBL, and they have been considered MBL-immunodeficient children according to several studies that have demonstrated associations between low-producing MBL-encoding alleles and an increased risk of infection in both children and adults.

These associations are strongest in infants at an age when passively acquired maternal immunity has decayed but the infant's adaptive immune repertoire is immature [12]. It has thus been suggested that MBL is of greatest importance when immune responses are either immature or defective.

All of the patients in the present study have only one thing in common: they all got sick with eosinophilic meningoencephalitis caused by *Angiostrongylus cantonensis*. The illness could have been produced from a possible accidental contamination with the larvae of the parasite, as this cause has been shown previously in other papers [9,13,14], together with a susceptibility to acquiring the parasitic infection because they were isolated cases. The patients reported here were living together with other children who were playing together with snails, but they were the only ones affected in spite of the fact that they were exposed to the same sanitary hygienic conditions.

It seems that MBL deficiency itself could not produce the illness, because the rest of the immune system components were not affected, according to work by previous researchers [11]. That is why we should establish an individual discussion of each of these patients who have MBL immunodeficiency.

It has been reported that MBL deficiency produces an increment of C4 [2]. It is possible that C4 could not be incorporated into the lectin pathway. Its accumulation could also produce a reduction of IgM levels. We should remember that IgM is the immunoglobulin class that more efficiently fixes the complement system because of its pentameric condition. It has been reported that IgA can fix complement system, and this class is also reduced.

It might be interesting to evaluate the concentrations of IgG subclasses in these patients in an attempt to deduce any possible association for subclasses deficiency [13]. In general, all the patients had an MBL immunodeficiency. The immune system tries to compensate with adequate IgG levels, but it cannot avoid the neurotoxic action of the larvae of *A. cantonensis *and the inflammatory process.

## Conclusions

There is no doubt that the study of MBL is of great importance and will help clinicians increase their knowledge of the immune response in patients with this parasitic illness on the basis of this case report, in which, for first time, this immune deficiency is reported in patients with *A. cantonensis*.

## Consent

Written informed consent was obtained from the parent or guardian of each patient for publication of this case series. Copies of the written consent are available for review by the Editor-in-Chief of this journal.

## Competing interests

The authors declare that they have no competing interests.

## Authors' contributions

BPD and AJDC designed and coordinated the study and drafted the manuscript. RBCF participated in its design and reviewed the clinical profiles of the patients. RHMA helped in the manuscript translation. HR and JCJ helped with protein analysis and in drafting the manuscript. All authors read and approved the final manuscript.
